# Arbuscular Mycorrhizal Symbiosis Imposes a Net Carbon Cost on Maize Under Phosphorus-Sufficient Conditions and Alters Nutrient-Dependent Scaling Trajectories

**DOI:** 10.3390/plants15121831

**Published:** 2026-06-12

**Authors:** Luqman Dau, Arunee Wongkaew, Wannasiri Wannarat, Worachart Wisawapipat, Kreingkrai Nonkum, Orawan Kumdee, Sirilak Kaewsuralikhit, Sutkhet Nakasathien

**Affiliations:** 1 Department of Agronomy, Faculty of Agriculture, Kasetsart University, 50 Ngamwongwan Road, Bangkok 10900, Thailand; luqmanramadhani.d@ku.th (L.D.); arunee.wo@ku.ac.th (A.W.); wannasiri.w@ku.th (W.W.); 2 Department of Soil Science, Faculty of Agriculture, Kasetsart University, 50 Ngamwongwan Road, Bangkok 10900, Thailand; worachart.w@ku.ac.th; 3 Kasetsart Agricultural and Agro-Industrial Product Improvement Institute, Kasetsart University, Bangkok 10900, Thailand; knonkum@ucdavis.edu; 4 Agricultural Research and Technology Transfer Center, Faculty of Agriculture, Kasetsart University, Bangkok 10900, Thailand; fagrowk@ku.ac.th; 5 Soil Microbiology Research Group, Soil Science Group, Agricultural Production Sciences Research and Development Division, Department of Agriculture, 50 Phahonyothin Road, Bangkok 10900, Thailand; sirilak@doa.in.th

**Keywords:** arbuscular mycorrhiza fungi, mycorrhiza-induced growth depression, optimal partitioning theory, root–shoot allometry, biomass allocation

## Abstract

The impact of arbuscular mycorrhiza fungi (AMF) on root–shoot scaling strategies under zinc and phosphorus deficiency remains poorly understood in maize. The aims of this study were (i) To quantify the effects of zinc/phosphorus deficiency on AMF colonization, (ii) to quantify biomass accumulation in different plant parts in the presence of AMF, and (iii) to characterize how AMF alter root–shoot allometric scaling under zinc/phosphorus deficiency. We conducted a pot experiment arranged in RCBD split plot with 6 replications. SUWAN 5819 maize seeds were grown for 22 days under five Hoagland’s solution-based nutrient regimes (+Zn+P, −Zn−P, +Zn−P, −Zn+P, and deionized water), with and without AMF. AMF colonization was highest (49.6%) under −Zn+P contrary to hypothesis 1 which predicted highest colonization under dual deficiency, while the deionized water treatment had the lowest colonization (30.1%). Phosphorus was the dominant factor affecting biomass accumulation with a 2–4-fold reduction in organ dry weights for phosphorus-deficient treatments compared to phosphorus-sufficient treatments. AMF colonization significantly reduced dry weights in +Zn+P by 8.6%, 19.0%, and 47.5% in the leaf, stem, and roots, respectively, consistent with mycorrhiza-induced growth depression (MGD). Nutrient deficiency resulted in root biomass accumulation, consistent with the optimal partitioning theory. AMF increased shoot mass fraction from 50% to 63% in +Zn+P, and from 41% to 52.5% in −Zn−P, suggesting AMF role in modulating biomass accumulation. Root–shoot scaling slopes derived from LMM revealed that zinc deficiency caused negative scaling trajectory, and AMF was associated with positive root–shoot scaling trajectory in the −Zn+P treatment, though the scaling relationship was not confirmed by SMA analysis. These findings highlight nutrient specific AMF-mediated growth dynamics in early vegetative stage.

## 1. Introduction

Phosphorus (P) and zinc (Zn) deficiencies each occur in 80% and over 50% of arable land globally, respectively, and co-occur across an estimated 30–40% worldwide, most notably in regions containing calcareous and tropical weathered soils [1,2,3,4,5]. In maize, these deficiencies severely affect photosynthetic capacity, hormonal signaling, and structural growth, resulting in substantial yield losses [6,7,8,9,10]. Although synthetic fertilizers can attenuate these effects, their use falls outside of sustainable agricultural practices, especially in the face of limited resources and global climate change [11,12].

Arbuscular mycorrhizal fungi (AMF) are obligate biotrophs from the subphylum Glomeromycotina that can form a symbiosis with the roots of approximately 80% of terrestrial plant species [13,14]. The symbiosis is initiated by the release of the plant hormones, strigolactones, which act as signals that diffuse into the rhizosphere and stimulate hyphal growth. In return, AMF release the lipochitooligosaccharide Myc factors that prepare the root for colonization [15,16]. Following hyphopodium formation, hyphae penetrate the cortex and differentiate into arbuscules, highly branched intracellular structures bounded by the plant-derived periarbuscular membrane (PAM) [15]. The membrane is an exchange interface between the plant and AMF for carbon derivatives from the host and nutrients (such as phosphorus and zinc) from the AMF [17,18]. When nutrient supply from soil is sufficient, however, mycorrhiza-induced growth depression (MGD) occurs where there is a net reduction in host biomass due to the symbiosis [19,20,21]. Understanding how AMF modulate plant growth as well as biomass allocation under zinc and phosphorus limitations is therefore essential for the correct use of AMF inoculants in nutrient-limiting environments.

Phosphorus is an essential macronutrient which serves as a structural constituent of nucleic acids, membrane phospholipids, and ATP [3]. Its deficiency triggers orthophosphate depletion in the chloroplast stroma which inhibits ATP synthase activity resulting in impaired photosynthetic electron transport chain [22]. Moreover, phosphorus deficiency compromises chlorophyll biosynthesis by disrupting magnesium chelatase function, which is ATP-dependent [23]. At the whole-plant level, phosphorus deficiency in maize results in stunted shoot growth, reduced leaf area, and a characteristic increase in the root-to-shoot ratio as the plant prioritizes belowground resources for scavenging nutrients [8,24].

Zinc is an essential micronutrient involved in more than 300 enzymatic reactions [2]. It plays roles in transcription factor structure functioning as a co-enzyme, RNA polymerase catalysis, and antioxidative defense as a structural part of superoxide dismutase [2,7]. In its role in plant growth, zinc is an obligate cofactor in the tryptophan biosynthetic pathway [25]. Tryptophan serves as the primary precursor for the synthesis of the plant hormone IAA, and so zinc deficiency leads to low tryptophan and IAA availability while accelerating IAA oxidative degradation by reactive oxygen species (ROS) [25,26]. The resulting IAA deficit results in stunted growth in both root and shoot tissue and could potentially result in growth imbalance between above- and belowground tissue.

According to the optimal partitioning theory (OPT), plants allocate biomass to the organ where resources are most limiting [27,28]. Despite a nutrient-limiting environment, plants have been observed to allocate biomass to the shoots in the presence of AMF, challenging the absolute applicability of this theory [29]. In studying biomass partitioning between roots and shoots, the static ratio shoot:root (S/R), which is often used as opposed to the dynamic allometric analysis, is the examination of how root–shoot scale is relative to one another as a plant grows [30]. No study, to our knowledge, has characterized how AMF alter root–shoot allometric scaling trajectories under zinc and phosphorus deficiency.

In this study we tested three hypotheses:

1. AMF colonization will be greatest under both zinc and phosphorus deficiency due to synergistic strigolactone-mediated signaling;
2. Under phosphorus-sufficient conditions, AMF will result in mycorrhiza-induced growth depression;
3. In nutrient deficiency, AMF will redirect biomass toward the shoot.

The objectives of this study were:

1. To quantify the effects of zinc and phosphorus deficiency on AMF colonization;
2. To quantify maize growth and biomass accumulation in different plant parts in the presence of AMF;
3. To characterize how AMF alter root–shoot allometric scaling under zinc and phosphorus deficiency.

## 2. Results

### 2.1. AMF Colonization

**Figure 1:**
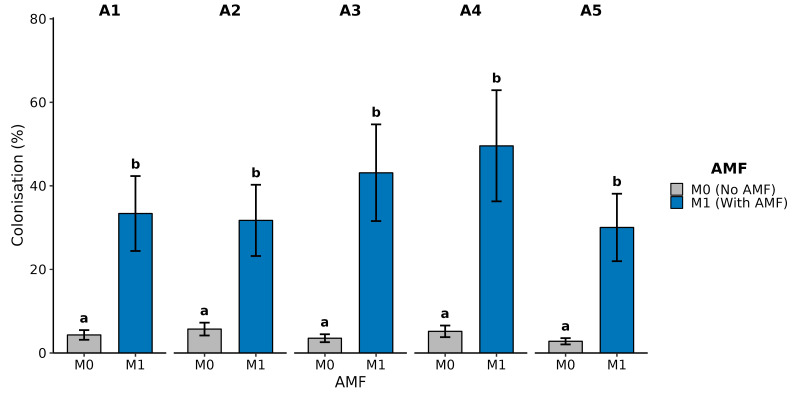
Effects of zinc and phosphorus deficiency combinations with and without arbuscular mycorrhiza fungi (AMF) inoculation on AMF root colonization (%) in maize. Bars represent back-transformed geometric means; error bars indicate SE of the back-transformed means. Grey bars = M0 (without AMF); blue bars = M1 (with AMF). Different lowercase letters within each nutrient treatment indicates significant differences by Sidak adjustment (*p* < 0.05). A1 = +Zn+P; A2 = −Zn−P; A3 = +Zn−P; A4 = −Zn+P; A5 = control.

There was a significant difference between inoculated and uninoculated treatments in AMF colonization (*p* < 0.001; Table 1; Figure 1). The lowest colonization percentage was from deionized water (30.1%), with the highest being from the treatment −Zn+P (49.6%), contrary to hypothesis 1. This is likely due to poor growth in AMF to establish a more robust symbiosis. Phosphorus-deficient treatments showed the highest colonization intensity, as reflected by high RCI values (Table 1). Nutrient treatments had no impact on AMF colonization (Table 1). The observed residual colonization in uninoculated pots (2.8–5.7%) likely reflect minor inoculant carryover. Moreover, the large significant difference between the inoculated and non-inoculated pots across all nutrient treatments (5–12 times higher M1 compared to M0) confirm that the residual colonization did not compromise data interpretation. It therefore does not represent functional symbiosis.

### 2.2. Chlorophyll Content (SPAD)

Phosphorus was the main determinant of chlorophyll content, with presence of phosphorus (+Zn+P and −Zn+P) resulting in high chlorophyll content, and its absence (−Zn−P, +Zn−P, and deionized water) resulting in low levels of chlorophyll (Table 2). Unlike phosphorous, zinc deficiency had no significant effect on chlorophyll content. In −Zn−P and +Zn−P treatments, uninoculated plants showed 13.2% and 9.7% higher chlorophyll values, respectively, than their inoculated counterparts. This indicates an AMF-associated carbon cost that reduces the chlorophyll content under phosphorus deficiency.

### 2.3. Specific Leaf Area and Leaf Area Ratio

Specific leaf area (SLA) was primarily determined by nutrient treatment (*p* < 0.001; Table 2), with no significant effect from AMF. The deionized water treatment produced the highest SLA (308–396 cm^2^ g^−1^), indicating thin leaves as an adaptation to severe nutrient deficiency. The −Zn−P treatment showed the lowest SLA (190–199 cm^2^ g^−1^) regardless of AMF status. LAR was significantly increased by AMF in the +Zn+P treatment (AMF: 112 cm^2^ g^−1^; no-AMF: 84.8 cm^2^ g^−1^; *p* < 0.05), observations that are consistent with greater shoot investment in the presence of AMF. LAR did not differ significantly between inoculation treatments in other nutrient combinations.

### 2.4. Biomass Accumulation and AMF Interaction

**Figure 2:**
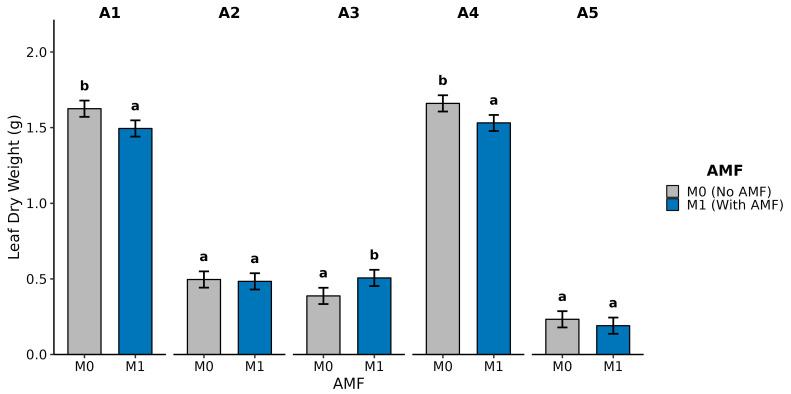
Effects of zinc and phosphorus deficiency with and without AMF on leaf dry weight in maize. Bars represent estimated marginal means; error bars indicate SE. Grey bars = M0 (without AMF); blue bars = M1 (with AMF). Different letters within each nutrient treatment indicate significant differences by Sidak adjustment (*p* < 0.05). A1 = +Zn+P; A2 = −Zn−P; A3 = +Zn−P; A4 = −Zn+P; A5 = control; M0 = without AMF; M1 = with AMF.

**Figure 3:**
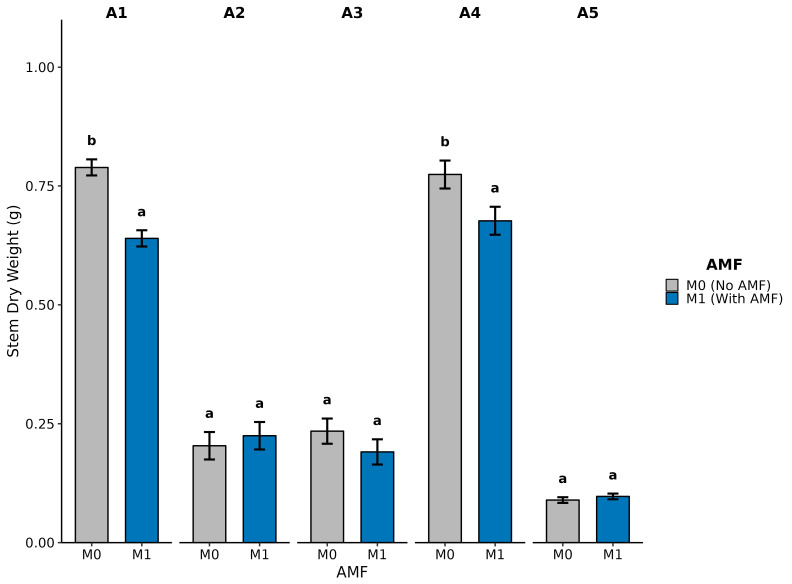
Effects of zinc and phosphorus deficiency with and without AMF on stem dry weight in maize. Bars represent estimated marginal means; error bars indicate SE. Grey bars = M0 (without AMF); blue bars = M1 (with AMF). Different letters within each nutrient treatment indicate significant differences by Sidak adjustment (*p* < 0.05). A1 = +Zn+P; A2 = −Zn−P; A3 = +Zn−P; A4 = −Zn+P; A5 = control; M0 = without AMF; M1 = with AMF.

**Figure 4:**
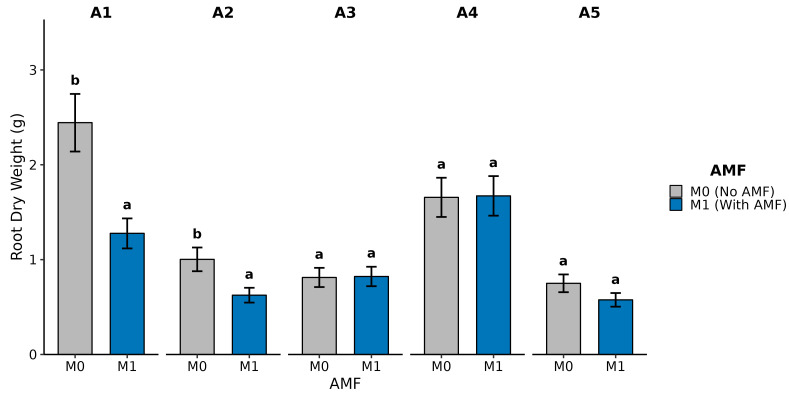
Effects of zinc and phosphorus deficiency with and without AMF on root dry weight in maize. Bars represent back-transformed geometric means from log-transformed data; error bars indicate SE. Grey bars = M0 (without AMF); blue bars = M1 (with AMF). Different letters within each nutrient treatment indicate significant differences by Sidak adjustment (*p* < 0.05). A1 = +Zn+P; A2 = −Zn−P; A3 = +Zn−P; A4 = −Zn+P; A5 = control; M0 = without AMF; M1 = with AMF.

Phosphorus was the dominant determinant of leaf, stem, and root dry weight across all treatments (*p* < 0.001; Table 3; Figure 2, Figure 3 and Figure 4). Treatments containing phosphorus (+Zn+P and −Zn+P) produced significantly greater biomass in all organs compared to phosphorus-deficient treatments.

The effect of AMF on dry weight accumulation was strongly dependent on nutrient treatment (Table 3). In +Zn+P, AMF most severely reduced root dry weight (47.5%), with less reductions in stem (19.0%) and leaf (8.6%) dry weights, resulting in total dry weight reduction of 29.7% (Table 3; Figure 2, Figure 3 and Figure 4). A similar pattern was observed in −Zn+P treatment where leaf and stem dry weights were reduced by 7.8% and 11.7%, respectively, though root dry weight was not significantly affected (Table 3). These results affirm hypothesis 2 and are consistent with mycorrhiza-induced growth depression (MGD) when nutrients are sufficient, specifically phosphorus. On the other hand, in the +Zn−P treatment, AMF significantly increased leaf dry weight by 23.5% relative to uninoculated plants (Figure 2), while stem and root dry weights were unaffected. This indicates that shoot-directed biomass accumulation was leaf-specific and was facilitated by AMF when phosphorus was the primary limiting resource.

### 2.5. Biomass Allocation Fractions

**Figure 5:**
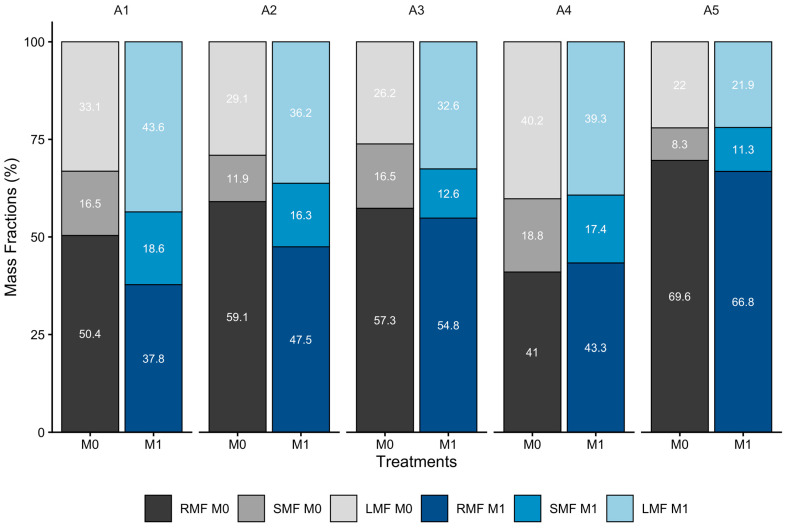
Effects of zinc and phosphorus deficiency with and without AMF on biomass allocation in maize. Each bar shows mean percentage contributions of root mass fraction (RMF; bottom, dark), stem mass fraction (SMF; middle), and leaf mass fraction (LMF; top, light) to total plant dry weight. Grey shades = M0 (without AMF); blue shades = M1 (with AMF). Values within segments show group mean percentages. A1 = +Zn+P; A2 = −Zn−P; A3 = +Zn−P; A4 = −Zn+P; A5 = control; M0 = without AMF; M1 = with AMF.

Root mass fraction (RMF) and shoot mass fraction were strongly determined by nutrient treatment (*p* < 0.001) and moderately by AMF (*p* < 0.05; Table 2; Figure 5). Plants in the deionized water treatment allocated 66–69% of total dry weight to roots, reflecting a strong belowground investment under severe nutrient limitation consistent with OPT. In the +Zn+P treatment, AMF presence significantly increased shoot mass fraction from 50% to 63%, specifically in the leaves (LMF: 34% to 44%), and decreased RMF from 50% to 37%. This indicates that the shift of biomass allocation towards the shoot in the presence of AMF occurs not only during nutrient deficiency but also in nutrient-sufficient conditions, specifically to the leaves, an affirmation of hypothesis 3. In the −Zn−P treatment, RMF decreased from 58% to 47% in the presence of AMF, indicating AMF modulation of OPT. No significant differences in biomass allocation were observed between inoculated and uninoculated plants in the remaining nutrient treatments.

### 2.6. Root–Shoot Allometric Scaling

**Figure 6:**
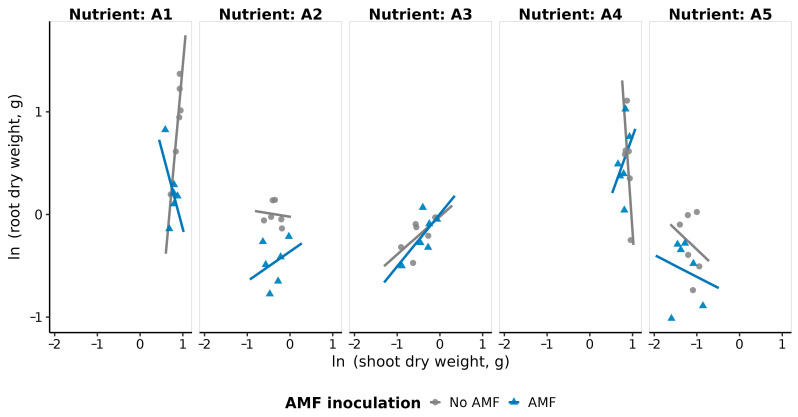
Root–shoot scaling relationships (ln root dry weight vs. ln shoot dry weight) in maize across five nutrient treatments with and without AMF inoculation. Lines represent group-specific scaling slopes derived from the linear mixed-effects model (Table 5). The most pronounced slope reversals occur in A1 (+Zn+P; slope reversal from +4.61 to −1.56) and A4 (−Zn+P; slope reversal from −6.06 to +1.16). A1 = +Zn+P; A2 = −Zn−P; A3 = +Zn−P; A4 = −Zn+P; A5 = control.

Root–shoot scaling slopes derived from linear mixed-effects model showed that root–shoot scaling was significantly influenced by nutrient treatments, AMF, as well as a three-way interaction with ln (shoot dry weight) across all nutrient levels relative to A1 (Table S3). In the +Zn+P treatment, root and shoot dry weights scaled positively in the absence of AMF (Table 4, Figure 6; slope = +4.61), indicating coordinated growth. However, the presence of AMF reversed the slope (Table 4, Figure 6; slope = −1.56), reflecting root growth suppression relative to shoot growth.

Zinc deficiency (−Zn+P) resulted in a negative trajectory in root–shoot scaling slope in the absence of AMF (slope = −6.06; Table 4, Figure 6). This indicates increasing shoot growth while root growth was suppressed. In the presence of AMF, a shift to positive root–shoot scaling trajectory was observed (slope = +1.16; Table 4, Figure 6). In the phosphorus-deficient treatments (−Zn−P, +Zn−P, deionized water), AMF produced only marginal slope improvements (Table 4), suggesting the dependency of AMF on the presence of phosphorus for a positive root–shoot scaling trajectory effect.

The standardized major axis (SMA) determined if there was a significant allometric relationship between root and shoot (p correlation) and how significantly different the slope (B) is from being isometric (p iso), where isometry is B = 1. The root–shoot allometric slopes derived from the (SMA) showed a significant correlation between root–shoot allometric scaling only in the +Zn+P without AMF treatment (Table 5; p corr = 0.009) with strong non-isometric scaling (Slope = 4.867; p iso = <0.001). These results indicate roots are outgrowing shoot when nutrients are sufficient in the absence of AMF, as well as the presence of coordinated growth between roots and shoot. The model did not detect a scaling relationship between the roots and shoot for the rest of the treatments (Table 5; p corr = ns). For treatments A1M1, A4M0, and A4M1, as the model did not detect any scaling relationship, this renders the slopes that are significant for non-isometry scaling irrelevant for interpretation within the SMA model.

### 2.7. Correlation Analysis and Principal Component

**Figure 7:**
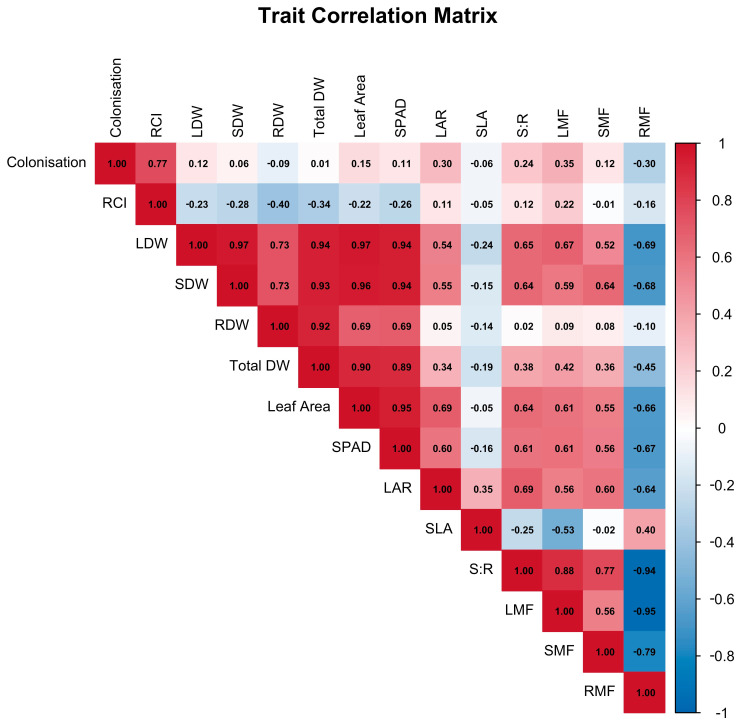
Pearson correlation matrix of 14 maize colonization, morphological, biomass, and allocation parameters. Color intensity and direction indicate the magnitude and sign of pairwise correlations (red = positive; blue = negative); numerical values are displayed within each cell. LDW = leaf dry weight; SDW = stem dry weight; RDW = root dry weight; total DW = total dry weight; leaf area = total leaf area; SPAD = leaf chlorophyll index; LAR = leaf area ratio; SLA = specific leaf area; S:R = shoot-to-root ratio; LMF = leaf mass fraction; SMF = stem mass fraction; RMF = root mass fraction; RCI = root colonization index.

The trait correlation matrix revealed a strong positive correlation (0.69–0.97) of the biomass traits and morphological traits (Figure 7): leaf dry weight (LDW), stem dry weight (SDW), root dry weight (RDW), total dry weight (Total DW), and leaf area. Leaf chlorophyll content (SPAD) was similarly positively correlated with biomass traits and shoot-related traits (0.60–0.97), consistent with coordinated investment in plant biomass and photosynthetic tissue. Leaf area ratio (LAR) showed moderate positive correlations with both shoot biomass and shoot allocation traits (0.34–0.69), reflecting it being a component of both specific leaf area and leaf mass fraction. The biomass allocation traits: leaf mass fraction (LMF), stem mass fraction (SMF), and shoot-to-root ratio (S/R), were also positively intercorrelated (0.88–0.77). Root mass fraction (RMF) was negatively correlated with all shoot allocation traits (−0.64 to −0.95) reaffirming a clear root–shoot allocation trade-off across treatments. AMF colonization and root colonization index (RCI) were strongly positively correlated with each other (0.77) but weakly correlated with most morphological traits (−0.06 to 0.30), suggesting that AMF colonization represents a largely independent axis of variation from overall plant morphology.

**Figure 8:**
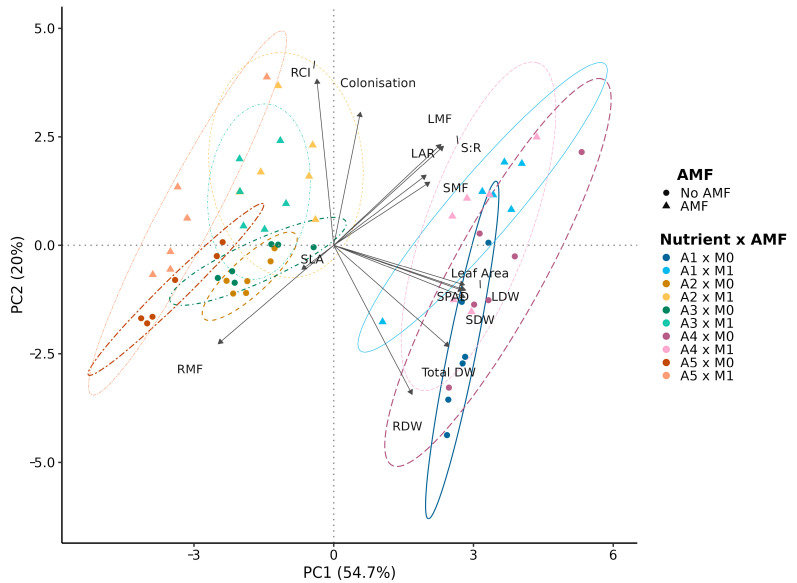
Principal component analysis biplot of 14 maize parameters showing PC1 (54.7%) against PC2 (20%). Circles = without AMF (M0); triangles = with AMF (M1). Solid ellipses represent 95% confidence regions for +Zn+P with AMF (dark blue) and +Zn+P without AMF (light blue). Dashed ellipses represent 95% confidence regions for each treatment combination with a nutrient deficiency. A1 = +Zn+P; A2 = −Zn−P; A3 = +Zn−P; A4 = −Zn+P; A5 = control; M0 = without AMF; M1 = with AMF.

In the PCA biplot, the first two principal components together explained 74.7% of the total variation in the dataset (PC1: 54.7%; PC2: 20%). The biplot vividly shows two cluster sets along PC1 based on biomass accumulation traits. The first is long arrows pointing to the positive (right-side) half of the biplot for total DW, LDW, SDW, leaf area, and SPAD, and the second is a long opposing arrow for RMF projecting to the negative (left-side) half (Figure 8). Plants on the positive side represent high biomass accumulation whereas those on the negative end have low biomass accumulation and high root investment for nutrient foraging. The two sets of clusters are differentiated by phosphorus sufficiency (positive) and phosphorus deficiency (negative) despite the presence of zinc with and without AMF along PC1.

PC2 was defined primarily by colonization and shoot allocation variables (colonization, RCI, LAR, S:R, LMF, SMF, RMF). Colonization variables and shoot allocation traits are projecting upwards with RMF projecting downwards towards the negative direction. Plants with positive PC2 scores showed shoot-biased allocation and high AMF colonization level, while plants with negative PC2 scores were root-dominant with low colonization.

The small angles between the biomass arrows (total DW, LDW, SDW, leaf area, SPAD) confirm strong positive correlations among these traits, consistent with the correlation matrix. The near 180° opposition between the shoot allocation cluster and RMF confirms the root–shoot allocation trade-off. RCI and Colonization projected nearly perpendicular to the biomass cluster, confirming statistical independence of AMF colonization from overall plant morphology.

## 3. Discussion

### 3.1. Phosphorus Drives Growth Responses While Zinc Governs Root–Shoot Scaling Dynamics

Our results show phosphorus as the principal determinant of maize biomass accumulation under the conditions tested, consistent with the status of phosphorus as the most production-limiting macronutrient in tropical agroecosystems [3]. Phosphorus deficiency disrupts the electron transport chain (via ATP synthase inhibition), impairs chlorophyll biosynthesis (by the absence of ATP necessary for Mg chelatase functionality), and suppresses auxin transport (by lack of PIN phosphorylation and ABC active transport inhibition). Collectively, these factors limit cell division, expansion, and photosynthate production [22,23,32]. These effects were pronounced in the 2–4-fold reduction in dry weights observed across all phosphorus-deficient treatments relative to their phosphorus-sufficient counterparts (Table 2; Figure 2, Figure 3 and Figure 4). This pattern of phosphorus-driven biomass divergence is reinforced by the multivariate analysis. The PCA revealed that biomass phenotypic variation among maize plants in this experiment accounted for 54.7% of phenotypic variation, largely under the influence of phosphorus (Figure 8). This reinforces the fundamental role of phosphorus in plant growth and overall biomass accumulation [3].

Zinc deficiency, despite having comparatively modest effects on total biomass, had a more pronounced effect on plant organ scaling dynamics. We hypothesize that the observed negative root–shoot scaling slope derived from LMM is linked to the essential role of zinc in IAA biosynthesis and stability [26,33,34]. Our results suggest that the root–shoot scaling consequences of zinc deficiency could be more severe to maize than total biomass reductions, a finding that point to the need for field studies in zinc-limited soils to arrive at more conclusive results in a broader setting for zinc-based fertilizer application.

### 3.2. AMF Imposes a Quantifiable Carbon Cost Under Nutrient Sufficiency

Root and stem dry weight reductions (47.5% and 19.0%) in +Zn+P in the presence of AMF provide evidence of mycorrhiza-induced growth depression in early vegetative stage (Table 2; Figure 4). MGD under nutrient sufficiency arises for mainly two reasons. First, the maintenance of the plant-derived periarbuscular membrane for the duration of the symbiosis. Second, in the symbiosis the plant continues to supply photosynthates and lipids to the fungus, thereby incurring a carbon cost [35,36]. Comparing the level of biomass reduction in the root (47.5%), stem (19.0%), and leaf (8.6%), it is evident that roots bear the primary carbon cost of sustaining the symbiosis when nutrients are non-limiting.

The elevated LAR in AMF-colonized +Zn+P plants (112 vs. 84.8 cm^2^ g^−1^; Table 3) further supports this interpretation. With less total biomass, AMF plants maintained relatively greater leaf area per unit mass, consistent with increased photosynthetic investment to support fungal carbon demand. These observations provide a direction for field studies to validate the potential occurrence of MGD in a field setting, the results of which could lead to more optimal application of AMF biofertilizers.

### 3.3. AMF Confers Leaf-Specific Phosphorus-Dependent Benefits Under Phosphorus Deficiency

Under phosphorus limitation (+Zn−P), AMF inoculation increased leaf dry weight by 23.5% without significantly affecting stem or root dry weight (Table 3). This selective enhancement of leaf biomass, the primary photosynthetic organ, aligns with OPT in the presence of AMF. When phosphorus is limiting, the fungal mycelium assumes the nutrient-scavenging role of roots, allowing the plant to reduce carbon investment at the roots and instead invest in photosynthetic tissue [29]. The absence of AMF benefits under dual zinc and phosphorus deficiency (−Zn−P) and the deionized water treatments is attributed to the level of severity caused by multi-nutrient stress. As AMF also require nutrients to function, the dual deficiency, especially of phosphorus, resulted in suppressed growth which may have impaired the extraradical mycelial network required for nutrient acquisition [37].

### 3.4. AMF Alter Root–Shoot Scaling in a Phosphorus-Dependent Manner

The results of our study have shown that AMF has a role in both static (S:R) and dynamic (root–shoot scaling) biomass allocation in early vegetative stage. AMF can change the direction of biomass allocation during growth depending on nutrient status (Table 4 and Table 5; Figure 6). The slopes derived from SMA for treatments A1M1, A4M0, and A4M1 showed significance in allometry but not in the scaling relationship itself. This rendered the slopes uninterpretable within the model, as allometry is dependent on the detection and presence of the scaling relationship first. The non-significant detection in the scaling relationship, despite significant slopes for allometry, could be due to the low number of observations (*n* = 6), which masked the interpretability of the slopes. However, the LMM provides a complementary analysis for the scaling relationship due to its statistical power advantage over the SMA.

Zinc deficiency was associated with a negative trajectory in root–shoot growth resulting in shoot-biased growth whereby the presence AMF resulted in a positive trajectory in root–shoot growth per the linear mixed-effects model (Table 4). We hypothesize that this change in slope trajectory is through the hormones strigolactones. Strigolactones have a role in stimulating hyphal growth towards the roots prior to symbiosis and are regulators of root architecture and lateral root branching [38,39]. The enhanced production of strigolactones in zinc deficiency could act as a compensation for the limited IAA production caused by the deficiency, thereby resulting in root development and a rebalancing in root–shoot scaling.

The shift in root–shoot scaling trajectory seems to be phosphorus-dependent, as positive root–shoot scaling was most effective in the treatments containing phosphorus (−Zn+P and +Zn+P), while phosphorus-deficient treatments showed slight AMF-mediated slope changes. This suggests that phosphorus availability could be necessary to observe the impact of AMF-mediated root–shoot scaling effects. The development of fungal structures, including extraradical and intraradical mycelium, is phosphorus dependent [37]. It has been observed by [37], that to maintain extraradical mycelium even after shoot removal, the AMF recapture previously supplied nitrogen and phosphorus from the host. The study further observed that AMF survival from host nutrients render them capable of establishing symbiosis with a different host. This study supports the importance of phosphorus for AMF function and survival, which could include AMF’s role in root–shoot scaling trajectory in a live host.

### 3.5. Limitations and Future Directions

There are some limitations to the study that limit the generalization of these conclusions. First, the duration of the experiment (22 days) reflects physiological and morphological responses in the early vegetative stage only. A study that captures plant responses in the reproductive stage is necessary to realize the full implications of biomass allocations and plant-AMF responses to yield. Second, pot experiments, being under a controlled environment, do not reflect the complex nature of field environments. Field studies account for biological and ecological complexities and influences of microbial communities in the soil, nutrient dynamics, soil physiochemical traits, and climatic variability. As they model real-world agronomic scenarios, it is essential to conduct the study under field conditions. Third, AMF responsiveness is genotype dependent [40]. Therefore, multi-genotype studies in maize are crucial to capture the full spectrum of the patterns observed. Fourth, nutrient treatments were applied as complete omission from Hoagland’s solution, representing extreme nutrient-deficient conditions than the moderate phosphorus and zinc deficiencies typical of agricultural soils. Caution is therefore necessary in extrapolating the magnitude of the observed AMF effects on scaling trajectories to field conditions where residual nutrient availability may partially sustain AMF function. Fifth, is the possible effect of inoculum carrier material which was not included in the M0 treatments. However, the carrier material represented 3% of the total substrate. Furthermore, the observed treatment effects were not consistent with carrier contribution. Therefore, the carrier material effect on nutrient availability is negligible.

Future research should focus on strigolactone, auxin, zinc, and phosphorus transport gene expression patterns across plant organs at multiple developmental stages. Integration of auxin and strigolactone quantification in colonized versus non-colonized roots under zinc deficiency would directly test the hormonal allometric stabilization hypothesis proposed here. Additionally, characterizing AMF allometric effects across multiple maize genotypes with higher sample size could reveal whether the scaling patterns observed here are broadly conserved or genotype-specific. Field studies incorporating realistic soil phosphorus and zinc gradients, diverse AMF communities, and measurements through the reproductive stage are ultimately required to assess whether AMF-mediated allometric stabilization under zinc deficiency translates into measurable yield benefits under agronomic conditions.

## 4. Materials and Methods

### 4.1. Location

The maize plants were grown for 22 days at the Central Lab greenhouse, Bangkhen campus, Kasetsart University (13 degrees 51′11.5″ N 100 degrees 34′20.4″ E).

### 4.2. Maize Seeds and Pot Preparation

The maize seeds used were from the maize variety SUWAN 5819, a single cross hybrid of Ki64 and Ki60, produced and provided by the Department of Agronomy, Kasetsart University, Bangkok, Thailand. Seeds were surface sterilized using a modified protocol [41]. In brief, the seeds were soaked in 10% analytical-grade sodium hypochlorite (*v*/*v*) for 15 min, rinsed 3 times with deionized water, placed on autoclaved germination paper, and incubated for 3 days at 25 °C (80% relative humidity). Upon germination, seedlings were transplanted into 1 kg acid-washed quartz sand in plastic pots.

### 4.3. Sand Washing

Sand was washed using a modified method by [42]. In summary, portions of 5 kg were first washed with tap water, soaked in 10% Clorox (2:1 sand:solution) for 2 h. Then they were rinsed 5 times with tap water and soaked in 0.1 M HCl for 24 h. After, they were rinsed 4 times with tap water followed by twice with RO water, and twice with deionized water to pH 7. The sand was then dried for 3 days, sieved (<1 mm), and autoclaved at 121 °C for 20 min.

### 4.4. Experimental Design and Treatments

The experiment was arranged as a randomized complete block split-plot design with six replications. Five levels of nutrient treatments were assigned to main plots, and 2 levels of AMF inoculation was assigned to sub-plots. Nutrient treatments were based on Hoagland solution had the following composition: 5 mM Ca(NO_3_)_2_.4H_2_O; 5 mM KNO_3_; 1 mM KH_2_PO_4_; 2 mM MgSO_4_.7H_2_O; 0.1 mM Fe-EDTA; 46 μM H_3_BO_3_; 9μM MnCl_2_.4H_2_O; 0.32 μM CuSO_4_.5H_2_O; 0.11 μM Na_2_MoO_4_.2H_2_O; 0.75 μM ZnSO_4_.7H_2_O. The solution was modified by the removal of KH_2_PO_4_ for −P treatments and removal of ZnSO_4_.7H_2_O (for −Zn treatments). The control treatment was only supplied with deionized water. The Hoagland’s solution pH was maintained and adjusted at 5.8–6.2 using drops of 4M NaOH.

Nutrient treatments commenced at 8 DAS. Soil moisture was maintained at 70% water-holding capacity via an automated timer to ensure each pot is supplied with the respective solution at the same time and equal volume. The nutrient treatments were designated: (A1) +Zn+P, (A2) −Zn−P, (A3) +Zn−P, (A4) −Zn+P, and (A5) deionized water. AMF inoculated treatments were designated (M1) and uninoculated (M0). The carrier material of the inoculant was a sterilized 1:1 mixture of sand: soil comprising of the species *Rhizophagus irregularis* with a propagule density of 25 spores/g supplied by the Soil Microbiology Research Group, Department of Agriculture, Thailand. Plants in M1 treatments received 30 g of the inoculant at 7 DAS. M0 treatments did not receive any inoculant and were instead an omission of inoculant material.

### 4.5. Sampling and Measurements

Destructive sampling was conducted at harvest (22 DAS). Leaf, stem, and root dry weights were obtained by drying fresh samples in a hot air oven at 70 °C for 48 h and weighing them after on an electric balance. Chlorophyll content was measured using a Konica Minolta SPAD-502Plus Chlorophyll Meter manufactured by Konica Minolta Sensing, Inc., Osaka, Japan. Each value was an average of 3 readings taken from the third fully expanded leaf (from the top) at 9 am [43]. Leaf area was calculated using the formula by [44]: Leaf area = leaf length × leaf width × 0.75. This correction has been previously applied to the variety SUWAN 5819 by [45] and was applied uniformly across all treatment groups.

### 4.6. Derived Growth and Allocation Parameters

Leaf area ratio (LAR), specific leaf area (SLA), leaf mass fraction (LMF), stem mass fraction (SMF), root mass fraction (RMF), shoot mass fraction, and shoot:root ratio were calculated as per [27].

### 4.7. AMF Colonization

Harvested root samples were stained with trypan blue [46] for visualizing colonization. The samples were first heated in KOH for ~7–10 min. They were then washed, stained, and left overnight. Colonization was quantified by the grid line intersection method at 45× magnification [47], and the root Colonization index (RCI), which is the measure of colonization intensity, was calculated as per [48].

### 4.8. Allometry

Root–shoot allometric relationships were examined using two complementary statistical methods. The first was a linear mixed-effects model (LMM) fitted using the lmer function from the lme4 (v2.0.1) package [49]. The model was specified as: ln(root DW) ~ ln(shoot DW) × nutrient × AMF + (1 | block) + (1|block:nutrient) where ln(root DW) and ln(shoot DW) are the natural logarithms of root and shoot dry weight respectively, applied to linearize the allometric power–law relationship. The fixed effects comprised the allometric predictor ln(shoot DW), the treatment factors nutrient (five levels), and AMF inoculation (two levels), and their fully saturated three-way interaction. This interaction structure allows the model to test whether the slope of the root–shoot scaling relationship differs depending on the combination of nutrient treatment and AMF inoculation status. The random effects comprised a random intercept for block (six replications) and a nested random intercept for block: nutrient. Group-specific root–shoot scaling slopes were derived from the fixed-effect coefficient estimates (Table S3) and are reported in Table 4.

The complementary method was standardized major axis (SMA) regression implemented in the smatr (v3.4.8) package [31]. Analyses were conducted across 10 treatment groups defined by the Nutrient × AMF interaction (*n* = 6 per group). Per-group SMA regressions were fitted independently for each nutrient × AMF combination to obtain group-specific allometric scaling exponents (B) and 95% confidence intervals, the significance of the bivariate scaling relationship (p_corr), and the significance of departure from isometry (p_iso). Isometry (B = 1) indicates proportional scaling; B > 1 indicates root mass increases disproportionately faster than shoot mass; B < 1 indicates shoot-dominant scaling. Per-group SMA are reported in Table 5.

Therefore, the LMM tests whether treatment factors significantly modified the root–shoot scaling slope across the full hierarchical dataset, while the SMA independently tests whether a significant bivariate allometric relationship exists within each treatment group and characterizes its exponent in biologically interpretable terms.

### 4.9. Statistical Analysis

All variables were analyzed by two-way ANOVA (*p* < 0.05) using the lmer function in the lme4 (v2.0.1) package. Normality of residuals and homogeneity of variance were verified using the Shapiro–Wilk test (*p* > 0.05) and Levene’s test (*p* > 0.05), respectively. Where normality assumptions were not met, log transformations were applied prior to analysis. Back-transformed geometric means and geometric standard deviations (GSD) are reported for these variables indicated by a dagger in Table 1, Table 2 and Table 3. Untransformed variables are reported as arithmetic means ± SD. Variables that violated homogeneity of variance with Levene’s test (stem dry weight and log-transformed specific leaf area) were analyzed with a heterogenous variance structure by the lme function with a varIdent weighting using the nlme (v3.1.169) package. The two functions (lmer and lme) were used to fit linear mixed-effects model (LMM) to account for the hierarchical experimental design of split plots using RStudio Version 2023.12.1+402. The fixed effects comprised of nutrient treatments (5 levels), AMF treatments (2 levels), and their interaction. Block (6 replications) was the random effect. All post-hoc pairwise comparisons were performed on the transformed scale using Sidak adjustment applied on estimated marginal means [50]. Compact letter displays in figures indicate significant pairwise differences at *p* < 0.05. Full ANOVA decomposition for all response variables is provided in Table S1 (Supplementary Materials).

Principal component analysis (PCA) was conducted to explore multivariate patterns of colonization (colonization and RCI), growth (LAR, SLA, leaf area, SPAD, leaf dry weight, stem dry weight, root dry weight, total dry weight) and biomass allocation (shoot: root, leaf mass fraction, stem mass fraction, root mass fraction). For PCA analysis and generation of the PCA biplot, two R packages, FactoMineR (v2.14) and factoextra (v2.0.0), were used. The number of components retained for interpretation was determined using two complementary criteria: the scree plot [51] and the Kaiser criterion [52] provided in Figure S1 (Supplementary Materials).

Pearson correlation matrix was analyzed using the R package corrplot (v0.95) [53] on colonization (colonization and RCI), growth (LAR, SLA, leaf area, SPAD, leaf dry weight, stem dry weight, root dry weight, total dry weight), and biomass allocation (shoot: root, leaf mass fraction, stem mass fraction, root mass fraction) parameters. All visualizations were produced using the ggplot2 (v4.0.3) [54] and ggrepel (v0.9.8) packages. GenAI claude (Sonnet 4.5, Anthropic, San Francisco, CA, USA) was used for assistance in data analysis and R code generation. Each line of code was manually validated by the authors for statistical appropriateness and reproducibility.

## 5. Conclusions

This study demonstrates that, in the early vegetative stage in a greenhouse setting, the agronomic value of AMF symbiosis in maize is dependent on the nutrient environment. Under phosphorus sufficiency, AMF imposed a significant carbon cost that resulted in MGD in both root and shoot. Under phosphorus limitation, AMF promoted leaf biomass accumulation, thereby increasing photosynthetic output for maintaining the symbiosis. Notably, AMF substantially altered the trajectory of root–shoot scaling when nutrients were non-limiting as well as when zinc was the limiting nutrient. In the former, AMF caused a negative trajectory in the scaling slope. In the latter, AMF caused a shift in the scaling trajectory from negative to positive under zinc deficiency in the presence of phosphorus. These findings give further insight into the role of AMF in not only shaping plant biomass accumulation but also dictating plant growth dynamics in the early vegetative stage.

## Figures and Tables

**Table 1:** Effect of zinc and phosphorus deficiency on AMF root colonization and root colonization index. Dagger (†): Colonization and RCI are geometric means from log-transformed data with 95% confidence intervals in parentheses. Different letters indicate significant differences by Sidak adjustment (*p* < 0.05). A1 = +Zn+P; A2 = −Zn−P; A3 = +Zn−P; A4 = −Zn+P; A5 = control; M0 = without AMF; M1 = with AMF; RCI = root colonization index.

Treatment	AMF	Colonization (%) †	RCI †
A1 (+Zn+P)	M0	4.31 (2.5–7.4) a	1.77 (0.9–3.3) a
	M1	33.38 (19.5–57.2) b	26.14 (14.0–48.7) b
A2 (−Zn−P)	M0	5.72 (3.3–9.8) a	5.70 (3.1–10.6) a
	M1	31.74 (18.5–54.4) b	50.70 (27.2–94.5) b
A3 (+Zn−P)	M0	3.53 (2.1–6.1) a	4.35 (2.3–8.1) a
	M1	43.15 (25.2–74.0) b	52.45 (28.1–97.8) b
A4 (−Zn+P)	M0	5.17 (3.0–8.9) a	3.12 (1.7–5.8) a
	M1	49.59 (28.9–85.0) b	29.65 (15.9–55.3) b
A5 (Control)	M0	2.80 (1.6–4.8) a	3.73 (2.0–7.0) a
	M1	30.05 (17.5–51.5) b	52.09 (27.9–97.1) b
Grand Mean		20.94	22.97
Nutrient		ns	ns
AMF		***	***
Nutrient × AMF		ns	ns

Signif. codes: 0 = ***; 0.1 = ns.

**Table 2:** Effects of zinc and phosphorus deficiency with and without AMF on growth and biomass distribution parameters. Dagger (†): leaf area, SLA and, shoot:root are geometric means (x/÷ GSD) from log-transformed data. SPAD, LAR, LMF, SMF, shoot MF and RMF are arithmetic means (+/− SD). A1 = +Zn+P; A2 = −Zn−P; A3 = +Zn−P; A4 = −Zn+P; A5 = control; M0 = without AMF; M1 = with AMF; LAR = leaf area ratio; SLA = specific leaf area; LMF = leaf mass fraction; SMF = stem mass fraction; RMF = root mass fraction; GSD = geometric SD.

Treatment	AMF	Leaf Area (cm^2^) †	SPAD	LAR (cm^2^ g^−1^)	SLA (cm^2^ g^−1^) †	Shoot:Root †	LMF	SMF	RMF
		Geo (x/÷ GSD)	Mean ± SD	Mean ± SD	Geo (x/÷ GSD)	Geo (x/÷ GSD)	Mean ± SD	Mean ± SD	Mean ± SD
A1 (+Zn+P)	M0	404.3 x/÷ 1.06	39.2 ± 1.22	84.8 ± 19.3	250.4 x/÷ 1.12	0.99 x/÷ 1.42	0.331 ± 0.05	0.165 ± 0.04	0.504 ± 0.09
	M1	370.6 x/÷ 1.27	38.9 ± 1.19	112 ± 31.5	249.7 x/÷ 1.18	1.66 x/÷ 1.46	0.436 ± 0.07	0.186 ± 0.03	0.378 ± 0.09
A2 (−Zn−P)	M0	97.6 x/÷ 1.07	26.5 ± 1.19	57.5 ± 5.08	199.2 x/÷ 1.24	0.69 x/÷ 1.24	0.291 ± 0.05	0.119 ± 0.01	0.591 ± 0.05
	M1	91.1 x/÷ 1.13	23.0 ± 3.72	69.0 ± 8.61	190.5 x/÷ 1.21	1.11 x/÷ 1.32	0.362 ± 0.05	0.163 ± 0.04	0.475 ± 0.07
A3 (+Zn−P)	M0	104.5 x/÷ 1.35	25.7 ± 1.18	75.1 ± 17.4	286.5 x/÷ 1.45	0.74 x/÷ 1.25	0.262 ± 0.05	0.165 ± 0.07	0.573 ± 0.05
	M1	95.5 x/÷ 1.20	23.2 ± 2.50	64.6 ± 14.4	197.9 x/÷ 1.46	0.82 x/÷ 1.22	0.326 ± 0.06	0.13 ± 0.02	0.54 ± 0.05
A4 (−Zn+P)	M0	428.8 x/÷ 1.04	40.2 ± 1.16	103 ± 14.6	258.7 x/÷ 1.05	1.47 x/÷ 1.62	0.401 ± 0.07	0.19 ± 0.04	0.40 ± 0.11
	M1	433.5 x/÷ 1.06	39.0 ± 1.70	113 ± 23.1	284.1 x/÷ 1.13	1.31 x/÷ 1.37	0.393 ± 0.05	0.174 ± 0.03	0.433 ± 0.08
A5 (Control)	M0	69.9 x/÷ 1.28	19.6 ± 2.03	68.8 ± 24.9	308.4 x/÷ 1.54	0.43 x/÷ 1.48	0.220 ± 0.08	0.083 ± 0.02	0.696 ± 0.08
	M1	70.9 x/÷ 1.43	20.1 ± 2.99	83.4 ± 19.0	395.9 x/÷ 1.62	0.49 x/÷ 1.55	0.219 ± 0.10	0.113 ± 0.02	0.668 ± 0.10
Grand Mean		216.75	29.5	83.1	262.13	0.97	0.32	0.15	0.53
Nutrient		***	***	***	***	***	***	***	***
AMF		ns	**	*	ns	*	**	ns	*
Nutrient × AMF		ns	ns	ns	ns	ns	ns	*	ns
CV/GCV (%)		19.1 †	6.3	23.2	14.2 †	35.1 †	17.8	24.0	15.1

Signif. codes: 0 = ***; 0.001 = **; 0.01 = *; 0.1 = ns.

**Table 3:** Effects of zinc and phosphorus deficiency with and without AMF on dry weight parameters of maize. LDW, SDW, and shoot DW are presented as arithmetic means +/− SD. Dagger (†): RDW and total DW are back-transformed geometric means from log-transformed data. Different letters within each nutrient treatment indicates Sidak adjustment (*p* < 0.05). A1 = +Zn+P; A2 = −Zn−P; A3 = +Zn-P; A4 = −Zn+P; A5 = control; M0 = without AMF; M1 = with AMF; LDW = leaf dry weight; SDW = stem dry weight; RDW = root dry weight; GSD = geometric SD.

Treatment	AMF	LDW (g)	SDW (g)	Shoot DW (g)	RDW (g) †	Total DW (g) †
		Mean ± SD	Mean ± SD	Mean ± SD	Geo Mean (x/÷ GSD)	Geo Mean (x/÷ GSD)
A1 (+Zn+P)	M0	1.63 ± 0.20 b	0.79 ± 0.03 b	2.42 ± 0.20 b	2.44 x/÷ 1.54 b	4.91 x/÷ 1.28 b
	M1	1.49 ± 0.18 a	0.64 ± 0.05 a	2.13 ± 0.21 a	1.28 x/÷ 1.38 a	3.45 x/÷ 1.13 a
A2 (−Zn−P)	M0	0.50 ± 0.09 a	0.20 ± 0.03 a	0.70 ± 0.11 a	1.00 x/÷ 1.12 b	1.70 x/÷ 1.09 b
	M1	0.48 ± 0.08 a	0.22 ± 0.09 a	0.71 ± 0.17 a	0.63 x/÷ 1.24 a	1.33 x/÷ 1.19 a
A3 (+Zn−P)	M0	0.39 ± 0.14 a	0.23 ± 0.09 a	0.62 ± 0.18 a	0.81 x/÷ 1.18 a	1.42 x/÷ 1.21 a
	M1	0.51 ± 0.16 b	0.19 ± 0.03 a	0.70 ± 0.18 a	0.82 x/÷ 1.23 a	1.51 x/÷ 1.25 a
A4 (−Zn+P)	M0	1.66 ± 0.11 b	0.77 ± 0.06 b	2.43 ± 0.13 b	1.66 x/÷ 1.56 a	4.18 x/÷ 1.17 a
	M1	1.53 ± 0.14 a	0.68 ± 0.09 a	2.21 ± 0.21 a	1.67 x/÷ 1.41 a	3.91 x/÷ 1.19 a
A5 (Control)	M0	0.23 ± 0.06 a	0.09 ± 0.02 a	0.32 ± 0.05 a	0.75 x/÷ 1.36 a	1.09 x/÷ 1.22 b
	M1	0.19 ± 0.08 a	0.10 ± 0.01 a	0.29 ± 0.08 a	0.58 x/÷ 1.38 a	0.87 x/÷ 1.25 a
Grand Mean		0.86	0.39	1.25	1.16	2.44
Nutrient		***	***	***	***	***
AMF		ns	ns	**	**	***
Nutrient × AMF		*	***	**	*	*
CV/GCV (%)		10.70	10.6	9.99	31.20 †	16.83 †

Signif. codes: 0 = ***; 0.001 = **; 0.01 = *; 0.1 = ns.

**Table 4:** Estimated allometric slopes (b) for the root–shoot ln–ln relationship (ln root dry weight ~ ln shoot dry weight) per Nutrient × AMF treatment group, derived from fixed-effect coefficients of the LMM (Table S3). Positive values indicate positive scaling of root and shoot; negative values indicate shoot growth with root growth suppression. A1 = +Zn+P; A2 = −Zn−P; A3 = +Zn−P; A4 = −Zn+P; A5 = Control.

Nutrient Treatment	AMF	Slope (b)	Interpretation
A1 (+Zn+P)	M0	4.61	Coordinated root–shoot growth
	M1	−1.56	AMF suppresses root relative to shoot
A2 (−Zn−P)	M0	−0.07	Dual deficiency decouples root–shoot allometry
	M1	0.28	Slight recovery: positive scaling trajectory with AMF under dual deficiency
A3 (+Zn−P)	M0	0.37	Weak positive: slower root growth
	M1	0.51	Slight increase: AMF modestly reinforces positive scaling under phosphorus deficiency
A4 (−Zn+P)	M0	−6.06	Strong negative: zinc deficiency causes extreme root suppression relative to shoot
	M1	1.16	strong positive scaling trajectory with AMF under zinc deficiency
A5 (Control)	M0	−0.40	Root growth suppressed with shoot growth
	M1	−0.22	Slight restoration by AMF but cannot reverse negative trajectory

Formula: b = B(ln shoot DW) + B(ln shoot × nutrient) + B(ln shoot × AMF) + B(ln shoot × nutrient × AMF) from Table S3 (Supplementary Materials).

**Table 5:** Standardized major axis (SMA) regression parameters for root–shoot allometry (ln root dry weight ~ ln shoot dry weight) fitted independently for each nutrient × AMF treatment group [31]. Slope (B): SMA allometric scaling exponent. 95% CI: confidence interval. Elevation: SMA y-intercept on the ln-ln scale. R^2^: squared Pearson correlation. p(corr.): significance of bivariate correlation. p(iso.): likelihood-ratio test of H0: slope = 1. *n* = 6 per group. A1 = +Zn+P; A2 = −Zn−P; A3 = +Zn−P; A4 = −Zn+P; A5 = control.

Nutrient Treatment	AMF	*n*	Slope (B)	95% CI	Elevation	R^2^	p(corr.)	p(iso.)
A1 (+Zn+P)	M0	6	4.867	[2.91, 8.13]	−3.383	0.851	0.009 **	<0.001 ***
	M1	6	−3.159	[−8.71, −1.15]	2.625	0.255	0.306 ns	0.030 *
A2 (−Zn−P)	M0	6	−0.714	[−2.17, −0.24]	−0.259	0.048	0.678 ns	0.520 ns
	M1	6	0.937	[0.31, 2.83]	−0.125	0.058	0.647 ns	0.900 ns
A3 (+Zn−P)	M0	6	0.586	[0.23, 1.53]	0.089	0.357	0.210 ns	0.235 ns
	M1	6	0.745	[0.31, 1.79]	0.096	0.487	0.123 ns	0.450 ns
A4 (−Zn+P)	M0	6	−8.287	[−20.79, −3.30]	7.871	0.423	0.162 ns	<0.001 ***
	M1	6	3.557	[1.23 10.33]	−2.289	0.150	0.448 ns	0.024 *
A5 (Control)	M0	6	−1.866	[−5.52, −0.63]	−2.415	0.108	0.525 ns	0.232 ns
	M1	6	−1.201	[−3.70, −0.39]	−2.078	0.012	0.838 ns	0.730 ns

Signif. codes: 0 = ***; 0.001 = **; 0.01 = *; 0.1 = ns.

## Data Availability

The data presented in this study are available upon request from the corresponding author.

## Supplementary Materials

The following supporting information can be downloaded at: https://www.mdpi.com/article/10.3390/plants15121831/s1, Table S1: Nutrient composition of Hoagland solution used; Table S2: Analysis of variance of growth and biomass parameters for zinc and phosphorus deficiency with and without mycorrhiza; Table S3: Fixed-effect parameter estimates from the linear mixed-effects model (LMM) fitted to ln(root dry weight) ~ ln(shoot dry weight) with nutrient (A1–A5; A1 = reference) and AMF inoculation (No AMF = reference) interactions. SE = standard error. Bold *p*-values: *p* < 0.05. Marginal R^2^: variance explained by fixed effects; conditional R^2^: variance explained by both fixed and random effects. A1 = +Zn+P; A2 = −Zn−P; A3 = +Zn−P; A4 = −Zn+P; A5 = control. Figure S1: scree plot with the Kaiser criterion for identifying principal components for the biplot.

## Abbreviations

The following abbreviations are used in this manuscript:

AMF	Arbuscular Mycorrhiza Fungi
RCBD	Randomized Complete Block Design
LMM	Linear Mixed Model
PAM	Periarbuscular Membrane
MGD	Mycorrhiza-induced Growth Depression
ATP	Adenosine Triphosphate
RNA	Ribonucleic Acid
IAA	Indole-3-acetic Acid
ROS	Reactive Oxygen Species
RO	Reverse Osmosis
DI	Deionized
LAR	Leaf Area Ratio
SLA	Specific Leaf Area
LMF	Leaf Mass Fraction
SMF	Stem Mass Fraction
RMF	Root Mass fraction
RCI	Root Colonization Index
SMA	Standardized Major Axis
PCA	Principal Component Analysis
ABC	ATP-Binding Cassette
