# Supplementary material for: Arbuscular Mycorrhizal Symbiosis Imposes a Net Carbon Cost on Maize Under Phosphorus-Sufficient Conditions and Alters Nutrient-Dependent Scaling Trajectories
Source: Plants (Basel). 2026 Jun 12;15(12):1831. doi: 10.3390/plants15121831 (PMC13306546; doi:10.3390/plants15121831)
Supplement: Supplementary file 1 [file plants-15-01831-s001.zip › plants-4341095-supplementary.pdf]

Supplementary Materials

Table S1: Nutrient composition of Hoagland solution used

| **Final concentration** | **WORKING SOLUTION** |
|---|---|
| **mM** | **STOCK A** |
| 5 | $Ca(NO_3)_2.4H_2O$ |
| 5 | $KNO_3$ |
| **mM** | **STOCK B** |
| 1 | $KH_2PO_4$ |
| **mM** | **STOCK C** |
| 2 | $MgSO_4.7H_2O$ |
| **mM** | **Fe STOCK** |
| 0.1 | Fe-EDTA |
| **µM** | **Micronutrient** |
| 46 | $H_3BO_3$ |
| 9 | $MnCl_2.4H_2O$ |
| 0.32 | $CuSO_4.5H_2O$ |
| 0.11 | $Na_2MoO_4.2H_2O$ |
| **µM** | **Zn STOCK** |
| 0.75 | $ZnSO_4.7H_2O$ |

Table S2: Analysis of variance of growth and biomass parameters for zinc and phosphorus deficiency with and without mycorrhiza

| Variable | Source of Variation | df | SS | MS | F-value | p-value | Significant codes |
|---|---|---|---|---|---|---|---|
| **AMF Colonisation (ln)** | Nutrient | 4 | 1.579 | 0.395 | 1.044 | 0.410 | **ns** |
| | AMF | 1 | 71.235 | 71.235 | 188.322 | < 0.001 | *** |
| | Nutrient × AMF | 4 | 1.148 | 0.287 | 0.759 | 0.562 | **ns** |
| **Leaf Dry Weight** | Nutrient | 4 | 7.122 | 1.781 | 209.170 | < 0.001 | *** |
| | AMF | 1 | 0.023 | 0.023 | 2.750 | 0.110 | **ns** |
| | Nutrient × AMF | 4 | 0.127 | 0.032 | 3.742 | 0.016 | * |
| **Stem Dry Weight †** | Nutrient | 4 | — | — | 479.448 | < 0.001 | *** |
| | AMF | 1 | — | — | 38.460 | < 0.001 | *** |
| | Nutrient × AMF | 4 | — | — | 10.882 | < 0.001 | *** |
| **Shoot Dry Weight** | Nutrient | 4 | 19.544 | 4.886 | 311.428 | < 0.001 | *** |
| | AMF | 1 | 0.126 | 0.126 | 8.059 | 0.009 | ** |
| | Nutrient × AMF | 4 | 0.286 | 0.072 | 4.564 | 0.007 | ** |
| **Root Dry Weight (ln) ‡** | Nutrient | 4 | 10.118 | 2.529 | 27.228 | < 0.001 | *** |
| | AMF | 1 | 1.113 | 1.113 | 11.976 | 0.001 | ** |
| | Nutrient × AMF | 4 | 1.026 | 0.256 | 2.761 | 0.038 | * |
| **Total Dry Weight (ln) ‡** | Nutrient | 4 | 14.008 | 3.502 | 125.430 | < 0.001 | *** |
| | AMF | 1 | 0.412 | 0.412 | 14.759 | < 0.001 | *** |
| | Nutrient × AMF | 4 | 0.312 | 0.078 | 2.792 | 0.048 | * |
| **Leaf Area (ln) ‡** | Nutrient | 4 | 29.556 | 7.389 | 205.606 | < 0.001 | *** |
| | AMF | 1 | 0.029 | 0.029 | 0.818 | 0.374 | **ns** |
| | Nutrient × AMF | 4 | 0.033 | 0.008 | 0.228 | 0.920 | **ns** |
| **SPAD** | Nutrient | 4 | 2,663.38 | 665.85 | 193.563 | < 0.001 | *** |
| | AMF | 1 | 28.70 | 28.70 | 8.344 | 0.008 | ** |
| | Nutrient × AMF | 4 | 32.17 | 8.04 | 2.338 | 0.083 | **.** |
| **Leaf Area Ratio** | Nutrient | 4 | 17,606.9 | 4,401.7 | 11.891 | < 0.001 | *** |
| | AMF | 1 | 1,691.7 | 1,691.7 | 4.570 | 0.037 | * |

| | | | | | | | |
|---|---|---|---|---|---|---|---|
| | Nutrient × AMF | 4 | 2,294.2 | 573.6 | 1.549 | 0.202 | **ns** |
| **Specific Leaf Area (ln) † ‡** | Nutrient | 4 | — | — | 2.588 | < 0.001 | *** |
| | AMF | 1 | — | — | 0.001 | 0.975 | **ns** |
| | Nutrient × AMF | 4 | — | — | 1.514 | 0.228 | **ns** |
| **Shoot:Root Ratio (ln) ‡** | Nutrient | 4 | 9.590 | 2.398 | 20.642 | < 0.001 | *** |
| | AMF | 1 | 0.753 | 0.753 | 6.481 | 0.014 | * |
| | Nutrient × AMF | 4 | 0.857 | 0.214 | 1.845 | 0.135 | **ns** |
| **Leaf Mass Fraction** | Nutrient | 4 | 0.169 | 0.042 | 12.700 | < 0.001 | *** |
| | AMF | 1 | 0.032 | 0.032 | 9.503 | 0.005 | ** |
| | Nutrient × AMF | 4 | 0.029 | 0.007 | 2.179 | 0.101 | **ns** |
| **Stem Mass Fraction** | Nutrient | 4 | 0.053 | 0.013 | 10.446 | < 0.001 | *** |
| | AMF | 1 | 0.001 | 0.001 | 0.859 | 0.358 | **ns** |
| | Nutrient × AMF | 4 | 0.014 | 0.003 | 2.737 | 0.039 | * |
| **Shoot Mass Fraction** | Nutrient | 4 | 0.525 | 0.131 | 20.718 | < 0.001 | *** |
| | AMF | 1 | 0.044 | 0.044 | 7.018 | 0.011 | * |
| | Nutrient × AMF | 4 | 0.049 | 0.012 | 1.953 | 0.116 | **ns** |
| **Root Mass Fraction** | Nutrient | 4 | 0.525 | 0.131 | 20.718 | < 0.001 | *** |
| | AMF | 1 | 0.044 | 0.044 | 7.018 | 0.011 | * |
| | Nutrient × AMF | 4 | 0.049 | 0.012 | 1.953 | 0.116 | **ns** |
| **Root Colonisation Index (ln) ‡** | Nutrient | 4 | 5.099 | 1.275 | 2.586 | 0.061 | **.** |
| | AMF | 1 | 90.153 | 90.153 | 182.851 | < 0.001 | *** |
| | Nutrient × AMF | 4 | 0.617 | 0.154 | 0.313 | 0.866 | **ns** |

AMF: Arbuscular Mycorrhiza Fungi; df: degree of freedom; SS: Sum Square; MS: Mean Square. Significance codes: *** $p < 0.001$; ** $p < 0.01$; * $p < 0.05$; . $p < 0.10$; ns: not significant. SS and MS are not available for models fitted with heterogeneous variance structure (lme with varIdent). † Stem Dry Weight and Specific Leaf Area: fitted with lme/varIdent; only F and p reported. (ln) ‡ represent variables that have been log-transformed prior to analysis.

Table S3: Fixed-effect parameter estimates from the linear mixed-effects model (LMM) fitted to ln(root dry weight) ~ ln(shoot dry weight) with Nutrient (A1-A5; A1=reference) and AMF inoculation (No AMF=reference) interactions. SE=standard error. Bold p-values: p<0.05. Marginal $R^2$: variance explained by fixed effects; Conditional $R^2$: variance explained by both fixed and random effects. A1=+Zn+P; A2=-Zn-P; A3=+Zn-P; A4=-Zn+P; A5=Control.

| Fixed Effect Term | Estimate (B) | SE | df | t | p-value |
|---|---|---|---|---|---|
| **(Intercept)** | -3.159 | 1.221 | 39.5 | -2.59 | 0.013 |

| | | | | | |
|---|---|---|---|---|---|
| **ln(Shoot DW)** | 4.613 | 1.384 | 39.6 | 3.33 | 0.002 |
| **A2** | 3.137 | 1.258 | 39.6 | 2.49 | 0.017 |
| **A3** | 3.140 | 1.246 | 39.6 | 2.52 | 0.016 |
| **A4** | 9.051 | 2.334 | 39.2 | 3.88 | <0.001 |
| **A5** | 2.421 | 1.485 | 39.9 | 1.63 | 0.111 |
| **AMF (inoculated)** | 4.582 | 1.386 | 19.9 | 3.31 | 0.004 |
| **ln(Shoot DW) x A2** | -4.680 | 1.581 | 39.9 | -2.96 | 0.005 |
| **ln(Shoot DW) x A3** | -4.242 | 1.450 | 39.7 | -2.93 | 0.006 |
| **ln(Shoot DW) x A4** | -10.674 | 2.628 | 39.2 | -4.06 | <0.001 |
| **ln(Shoot DW) x A5** | -5.008 | 1.567 | 39.8 | -3.20 | 0.003 |
| **ln(Shoot DW) x AMF** | -6.176 | 1.661 | 19.1 | -3.72 | 0.001 |
| **A2 x AMF** | -4.922 | 1.439 | 21.8 | -3.42 | 0.002 |
| **A3 x AMF** | -4.558 | 1.417 | 20.0 | -3.22 | 0.004 |
| **A4 x AMF** | -10.871 | 2.597 | 30.6 | -4.19 | <0.001 |
| **A5 x AMF** | -4.666 | 1.687 | 21.2 | -2.77 | 0.012 |
| **ln(Shoot DW) x A2 x AMF** | 6.533 | 1.927 | 27.7 | 3.39 | 0.002 |
| **ln(Shoot DW) x A3 x AMF** | 6.316 | 1.755 | 19.4 | 3.60 | 0.002 |
| **ln(Shoot DW) x A4 x AMF** | 13.394 | 3.026 | 29.9 | 4.43 | <0.001 |
| **ln(Shoot DW) x A5 x AMF** | 6.358 | 1.848 | 20.1 | 3.44 | 0.003 |
| **Model Fit** | | | | | |
| **Marginal $R^2$ (fixed effects only)** | 0.760 | | | | |
| **Conditional $R^2$ (fixed + random)** | 0.808 | | | | |
| **Shapiro-Wilk (residuals)** | W = 0.990, p = 0.897 (normally distributed) | | | | |

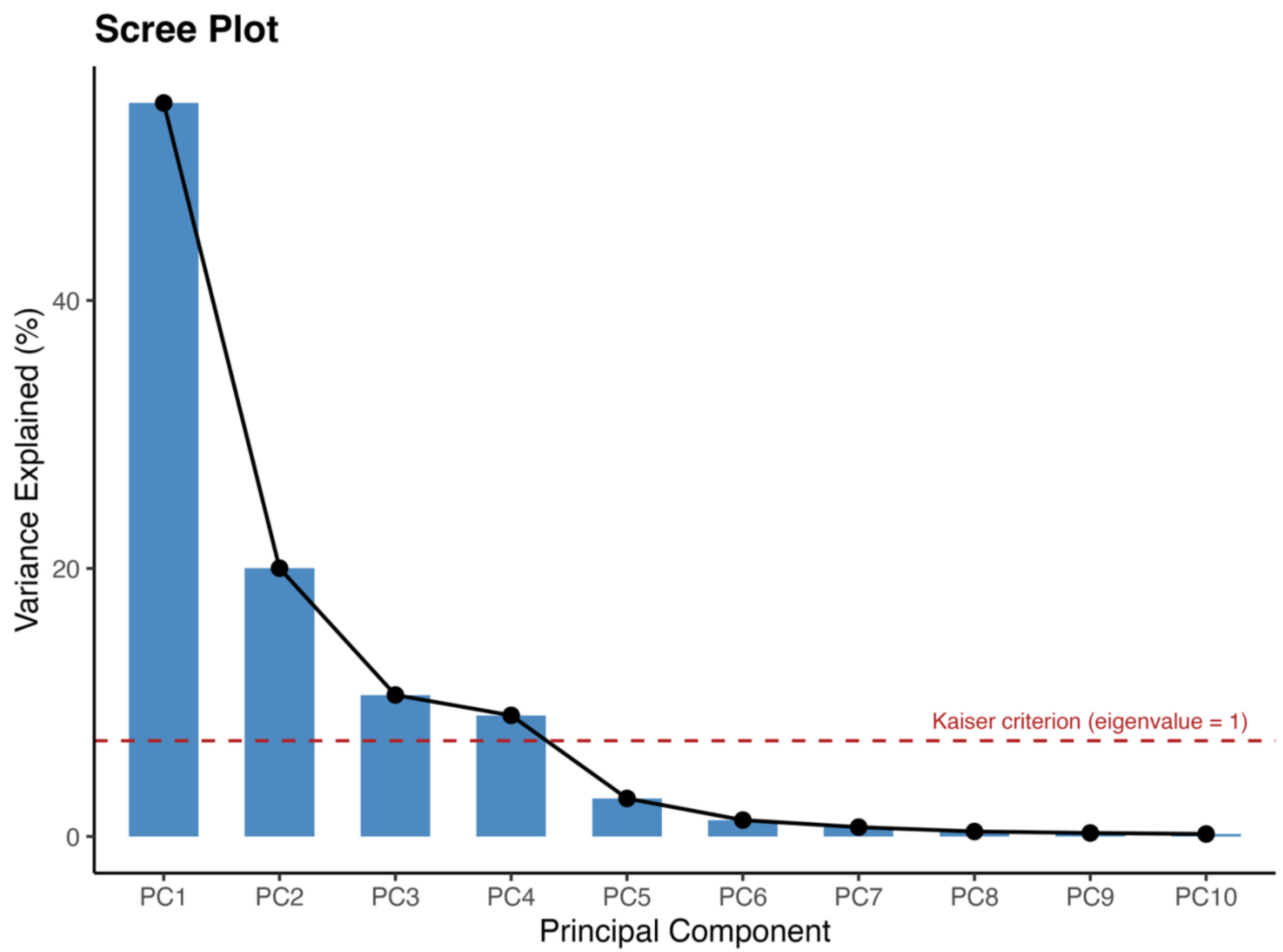


Figure S1: Scree plot with the Kaiser criterion for identifying principal components 1 and used for the biplot.
